# Transcriptional Regulation by CHIP/LDB Complexes

**DOI:** 10.1371/journal.pgen.1001063

**Published:** 2010-08-12

**Authors:** Revital Bronstein, Liron Levkovitz, Nir Yosef, Michaela Yanku, Eytan Ruppin, Roded Sharan, Heiner Westphal, Brian Oliver, Daniel Segal

**Affiliations:** 1 Department of Microbiology and Biotechnology, Tel Aviv University, Tel Aviv, Israel; 2 Department of Physiology and Pharmacology, Tel Aviv University, Tel Aviv, Israel; 3 Balvatnik School of Computer Science, Tel Aviv University, Tel Aviv, Israel; 4 Section on Mammalian Molecular Genetics, Program in Genomics of Development, Eunice Kennedy Shriver National Institute of Child Health and Human Development, National Institutes of Health, Bethesda, Maryland, United States of America; 5 Laboratory of Cellular and Developmental Biology, National Institute of Diabetes and Digestive and Kidney Diseases, National Institutes of Health, Bethesda, Maryland, United States of America; Harvard Medical School, Howard Hughes Medical Institute, United States of America

## Abstract

It is increasingly clear that transcription factors play versatile roles in turning genes “on” or “off” depending on cellular context via the various transcription complexes they form. This poses a major challenge in unraveling combinatorial transcription complex codes. Here we use the powerful genetics of *Drosophila* combined with microarray and bioinformatics analyses to tackle this challenge. The nuclear adaptor CHIP/LDB is a major developmental regulator capable of forming tissue-specific transcription complexes with various types of transcription factors and cofactors, making it a valuable model to study the intricacies of gene regulation. To date only few CHIP/LDB complexes target genes have been identified, and possible tissue-dependent crosstalk between these complexes has not been rigorously explored. SSDP proteins protect CHIP/LDB complexes from proteasome dependent degradation and are rate-limiting cofactors for these complexes. By using mutations in SSDP, we identified 189 down-stream targets of CHIP/LDB and show that these genes are enriched for the binding sites of APTEROUS (AP) and PANNIER (PNR), two well studied transcription factors associated with CHIP/LDB complexes. We performed extensive genetic screens and identified target genes that genetically interact with components of CHIP/LDB complexes in directing the development of the wings (28 genes) and thoracic bristles (23 genes). Moreover, by *in vivo* RNAi silencing we uncovered novel roles for two of the target genes, *xbp1* and *Gs-alpha*, in early development of these structures. Taken together, our results suggest that loss of SSDP disrupts the normal balance between the CHIP-AP and the CHIP-PNR transcription complexes, resulting in down-regulation of CHIP-AP target genes and the concomitant up-regulation of CHIP-PNR target genes. Understanding the combinatorial nature of transcription complexes as presented here is crucial to the study of transcription regulation of gene batteries required for development.

## Author Summary

Different cell types in multi-cellular organisms are determined by the repertoire of genes active in each cell. This repertoire, or transcriptome, is established by the coordinated activity of transcription factors and cofactors that form modular transcription complexes. The modular nature of transcription complexes complicates our understanding of how transcription factors shape the transcriptome. CHIP/LDB transcription complexes direct formation of various cell types including blood and nerve cells. CHIP/LDB malfunction leads to developmental defects and cancer. The function of these complexes depends critically on the docking of specific transcription factors and co-factors at a specific time and in a specific cell type, making them outstanding models for intricate transcriptional regulation. Here we demonstrate that loss of SSDP, a key regulatory component of CHIP/LDB transcription complexes, alters transcription of a large group of genes. We used bioinformatics tools and genetic tests to examine the function of additional components of CHIP/LDB transcription complexes and their target genes during the development of specific organs. We demonstrate how differences in the availability of transcription factors in different cells can affect the function and composition of CHIP/LDB transcription complexes.

## Introduction

The intricate regulation of gene expression in multi-cellular organisms involves an elaborate collaboration between repertoires of cis-regulatory sequences and modular, multi-protein transcription complexes that bind them (reviewed in [1]). Transcription complexes are now viewed as being composed of relatively ubiquitous core elements and a variety of context-dependent cofactors that interact with the core elements to regulate context-specific transcription (reviewed in [2]). An increasing number of such cofactors are being identified and the diverse roles of each transcription complex is thought to depend on the unique combination of associated cofactors (reviewed in [1]–[3]).

**Figure 1:**
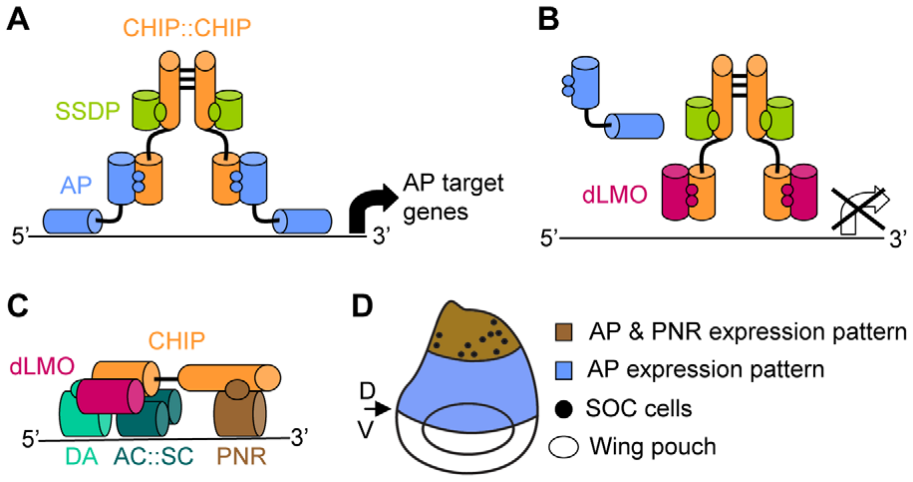
Composition and function of the CHIP-AP and CHIP-PNR transcription complexes. (A) The CHIP-AP complex is composed of a dimer of CHIP molecules bound through their dimerization domain [7]. Each molecule of CHIP can bind one molecule of AP [8], [9] through its LIM interacting domain (LID) [7], [8] and one molecule of SSDP through its LDB/CHIP conserved domain (LCCD) [10]. (B) dLMO displacement of AP from the complex blocks AP dependant expression of target genes [9]. (C) The CHIP-PNR complex is composed of a single CHIP molecule that binds dLMO through its LID domain. PNR binds CHIP in a region that overlaps CHIP's dimerization domain thus preventing the formation of a CHIP dimmer. The b-HLH members of this complex are the AC:SC heterodimer and the DA protein [16]. (D) Schematic representation of the wing imaginal disc (dorsal side is up). AP expression in the dorsal area (in blue) determines the dorsal compartment. The boundary between AP expressing and non-expressing cells determines the dorsal (D) ventral (V) boundary which will give rise to the adult wing margin. The wing poach will give rise to the adult wing blade. PNR expression in the dorsal most area (in brawn) of the wing imaginal disc determines thoracic identity. The SOC cells that will give rise to the thoracic macrocheata are indicated by dots.

A prime example for this combination of general and specific factors are complexes formed by transcription factors that interact with cofactors of the CHIP/LDB family. CHIP is a *Drosophila* gene product that is closely related to the LDB (alias CLIM or NLI) proteins that have been well preserved in evolution all the way from *Caenorhabditis elegans* to man. These multi-adaptor proteins mediate interactions between different classes of transcription factors and additional co-regulators of transcription (reviewed in [4]). One of the best studied CHIP/LDB complexes is the *Drosophila* CHIP-APTEROUS complex (Figure 1A). APTEROUS (AP) is a LIM-homeodomain (LIM-HD) transcription factor [5] homologue of mammalian LHX2 and LHX9 [6]. The CHIP-AP complex is composed of a dimer of CHIP molecules [7], each of which binds one molecule of AP [8], [9] through a LIM interacting domain (LID) [7], [8] and one molecule of single-stranded DNA-binding protein (SSDP) through a CHIP/LDB conserved domain (LCCD) [10]. In the fly, this complex triggers a signaling cascade that specifies the dorsal compartment of the wing imaginal disc and serves to define the dorsal/ventral boundary at the adult wing margin (reviewed in [11]).

CHIP-AP complex function is negatively regulated by the *Drosophila* LIM-only (dLMO) protein (Figure 1B). dLMO binds CHIP *in vitro* and competes with AP for binding to CHIP [9]. This cofactor exchange is crucial for the proper function of the CHIP-AP complex during wing imaginal disc development as evident from the analysis of mutant and transgenic flies [7]–[9], [12]–[15].

An additional level of regulation is introduced by concomitant protein-protein interaction and cofactor exchange with non-LIM transcription factors (Figure 1C). Specifically, CHIP and dLMO form an alternative complex together with a GATA family transcription factor, PANNIER (PNR), and the beta-HLH transcription factors ACHAETE (AC), SCUTE (SC), and DAUGHTERLESS (DA) [16]. We refer to this complex as CHIP-PNR. One function of the CHIP-PNR complex is directed toward thoracic macrochaete (sensory bristles) differentiation (Figure 1D). The pattern of sensory bristles reflects the distribution of precursor sensory mother cells in the wing imaginal disc. These precursors are specified during the third larval instar and early pupal stages from a restricted group of cells that express *ac* and *sc* [17]. The expression of *ac* and *sc*, in turn, is regulated in part by the CHIP-PNR complex [16].

In the context of the CHIP-PNR complex, dLMO is a positive regulator [18], [19] and DNA binding is mediated through the GATA and beta-HLH transcription factors. There is a complex antagonistic relationship between CHIP-PNR and CHIP-AP, as the interaction between CHIP and PNR prevents CHIP from forming the homodimer that is crucial for the function of the CHIP-AP complex. Indeed, the function of the CHIP-PNR complex is antagonized by AP [16].

Like the CHIP/LDB encoding genes themselves, the components, assembly, and function of CHIP/LDB-based complexes appears to be highly conserved [6], [13], [20], [21]. For example, complexes containing SSDP1, LDB1 and LHX2 or LHX3 (termed LDB-LHX) are found in the mouse pituitary cell line alfaT3-1 [22], and a complex containing LDB1, GATA-1, LMO2, TAL1 and E47 (termed LDB-GATA) regulates erythropoiesis in mice [23]-[25].

SSDP proteins play a crucial role in the formation, stability and function of CHIP/LDB-based complexes in flies and mice [10], [13]. SSDP proteins promote assembly of LDB-LHX and LDB-GATA complexes and contribute to their transcription activity. Moreover, proteasome-mediated turnover of LDB1, LHX and LMO proteins is inhibited by formation of a complex with SSDP proteins [22], [26], [27]. Thus, the functional interaction between LDB and SSDP proteins appears to be independent of the specific composition of LIM or non-LIM proteins within the complex.

While the function of CHIP/LDB complexes depends on SSDP, the function of SSDP proteins in turn depends on interaction with CHIP/LDB complexes: both in flies and in mammals SSDP proteins do not contain a nuclear localization signal and have to bind CHIP/LDB in order to enter the nucleus [10]. Thus, SSDP proteins are key components of CHIP/LDB complexes in both functionality and specificity. CHIP/LDB and SSDP are therefore a valuable model for studying the intricacies of transcriptional regulation at the genomic level. Here we address genome-wide effects of *Drosophila* SSDP on the transcriptional activity of CHIP/LDB-based complexes. Using a combination of microarray analysis and genetic interaction tests we identified novel genes downstream of SSDP that affect the development of wing and thoracic bristle development. Using transcription factor binding site analysis, we were able to show that SSDP makes distinct contributions to the transcriptional activity of the CHIP-AP and the CHIP-PNR complex.

## Results

### Expression profiles of SSDP mutants

We have conducted a genomic search for putative SSDP target genes using *Drosophila* microarrays [28] to report expression of 14,142 predicted transcripts. Poly-A^+^ RNA was extracted from third instar larvae (males only to avoid potentially confounding sex-biased gene expression). We used two different heteroallelic combinations of *ssdp* hypomorphic alleles, *ssdp^neo48^/ssdp^BG1663^* and *ssdp^31^/ssdp^BG1663^*, which allow survival up to the pupal stage [13]. We opted to use heteroallelic combinations of *ssdp* on different genetic backgrounds rather than homozygotes, in order to minimize inadvertent homozygosity for extraneous mutations. The heteroallelic mutant pairs were compared to each of the corresponding single heterozygotes (Table S1). We identified 189 candidate target genes that were differentially expressed between experimental and control samples (FDR corrected p<0.05; Table S2). Since SSDP is believed to be a positive transcriptional regulator of the CHIP/LDB complex [10], [13], we expected most of the target genes to exhibit lower expression in the *ssdp* mutants compared to the heterozygous controls. Interestingly, only a third of the 189 genes met this expectation (Table S2). These results might suggest that SSDP has a hitherto unidentified negative transcriptional regulatory effect on certain genes. Alternatively, secondary targets may be negatively regulated by direct targets of SSDP. 

### The upstream regulatory sequences of SSDP target genes are enriched for binding sites of bona-fide CHIP/LDB-associated transcription factors

**Figure 2:**
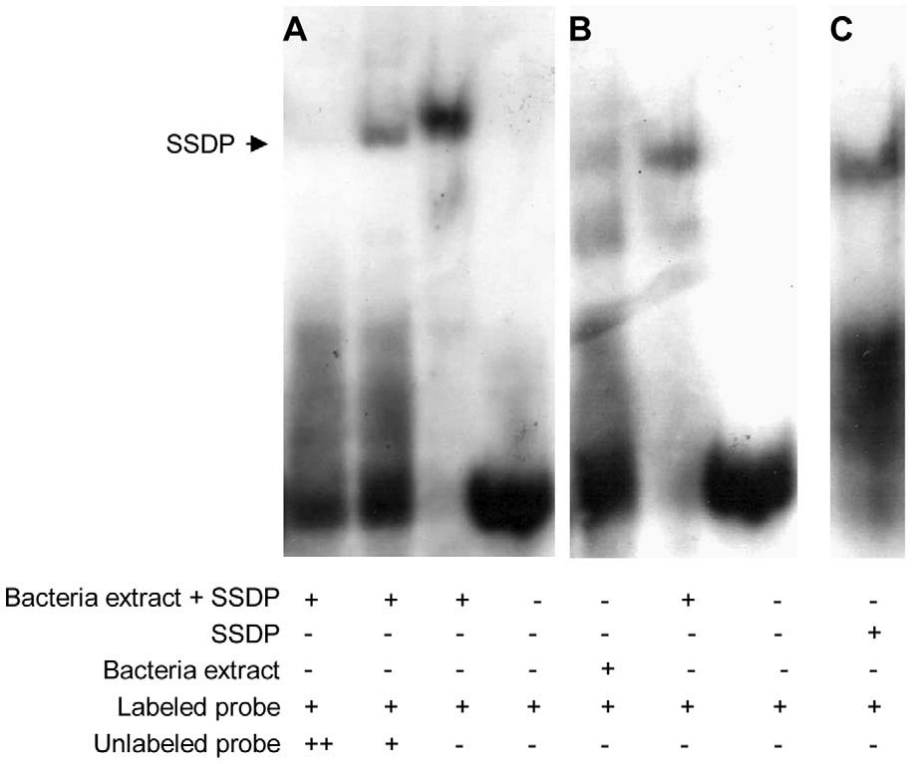
Fly SSDP specifically recognizes the SSDP binding site. (A) Labeled single stranded oligonucleotide representing the binding site of chicken SSDP was incubated with or without cell extracts from bacteria expressing GST-tagged fly SSDP in the presence of increasing concentrations of unlabeled oligonucleotide as a competitor. (B) Labeled single stranded binding site of chicken SSDP was incubated with cell extracts from bacteria expressing or not expressing GST-tagged fly SSDP. (C) Labeled single stranded binding site of chicken SSDP was incubated with purified GST-tagged fly SSDP. B and C were taken from the same gel.

One way of testing for direct targets of SSDP is to look for enrichment for SSDP binding sites in the upstream regions of the 189 putative target genes. SSDP was first identified due to its ability to bind a single stranded poly-pyrimidine sequence present in the promoter of the chicken alfa-2(I) collagen gene [29]. Our gel shift experiments showed that this binding site is specifically recognized by fly SSDP (Figure 2).

We searched for enrichment for putative SSDP binding sites in the 500 bp upstream region of the 189 candidate genes identified in the microarray work, using two algorithms, PRIMA [30] and DEMON, We found SSDP binding site enrichment upstream the 189 candidate genes (p = 0.037) using DEMON. Interestingly, the SSDP binding site was even more significantly enriched (p = 0.02) among the genes down-regulated in the mutants, while there was weak significance among the genes up-regulated in the mutants (p = 0.17). This is consistent with the accepted role for SSDP as a positive transcriptional regulator. These data suggest that a significant number of the genes down-regulated in mutants are indeed direct targets of SSDP.

In order to determine whether SSDP target genes are also likely CHIP/LDB target genes, we searched the same upstream regions for binding sites of AP and PNR, transcription factors known to function in the CHIP/LDB complex. Binding site matrices for all available insect transcription factors (including the AP binding site) were obtained from TransFac [31], and a matrix of PNR binding sites, not included in TransFac, was constructed [32]. Strikingly, the PRIMA algorithm detected impressive enrichment for both AP (p = 0.04) and PNR (p = 4.64E-07) binding sites. Enrichment for the latter was also detected by DEMON (p = 2.64E-05). Interestingly, the enrichment for the AP binding site was lost when the down- (PRIMA: p = 0.085 and DEMON: p = 0.67) and up- regulated (PRIMA: p = 0.33 and DEMON: p = 0.21) gene groups were analyzed separately. This suggests that both groups harbor genes that are targeted by AP. The enrichment for SSDP and AP binding sites in the genes down-regulated in *ssdp* mutants is in agreement with SSDP functioning as a positive cofactor of the CHIP-AP complex. In contrast, the PNR binding sites were significantly enriched in the genes up-regulated in the mutants (PRIMA: p = 2.59E-05 and DEMON: p = 6.1E-06) but not in the genes down-regulated in the mutants (PRIMA: p = 0.085 and DEMON: p = 0.27). This suggests that a significant number of the genes up-regulated in *ssdp* mutants are direct targets of PNR. Given that AP and PNR bind to CHIP competitively during *Drosophila* thorax formation [16], we suggest that loss of SSDP disrupts the normal balance to favor CHIP-PNR complex formation. This would result in the down-regulation of CHIP-AP target genes and the simultaneous up-regulation of the CHIP-PNR target genes. Furthermore, up-regulated AP target genes may be regulated by both complexes. For example, AP and PNR are both known to positively regulate the expression of *stripe*, a key gene regulating development of the wing imaginal disc [33], [34].

In addition to the expected enrichment for the SSDP, AP and PNR binding sites upstream of the candidate target genes, we found enrichment for several other binding sites in the upstream regions of these genes (see Table S3 for p-values and binding sites information). Whether the function of all of these transcription factors is dependent on, or independent of, SSDP and/or of the CHIP/LDB transcription complexes remains to be determined. However, several of them have already been implicated in CHIP/LDB complex function (see Discussion).

The fact that the 189 putative SSDP target genes identified in our microarray experiments are enriched for binding sites of SSDP itself and its known partners in transcription is an independent orthogonal validation of the microarray results. These data encouraged us to ask if these putative targets have a genetic function in developmental events mediated by CHIP/LDB.

### SSDP target genes interact genetically with the CHIP/LDB transcription cofactor dLMO

The analysis of SSDP target genes suggested that they are targeted by both CHIP-AP and CHIP-PNR complexes. To simplify the interpretation of genetic tests, we chose to begin looking for functional interactions between SSDP target genes and the CHIP-AP complex in the wing, where *pnr* is not expressed [35].

In the wing imaginal disc the CHIP-AP complex is involved in determination of the dorsal compartment. The edge of the CHIP-AP domain is the dorsal/ventral (D/V) boundary which will later give rise to the adult wing margin. Subtle disruption of the transcription activity of the CHIP-AP complex causes irregularities in the D/V boundary, which are evident as notches in the adult wing margin [12], [36], [37]. Indeed, such disruptions occur in the over-expression allele, *Dlmo^Bx^* which encodes a negative regulator of the CHIP-AP complex.

**Figure 3:**
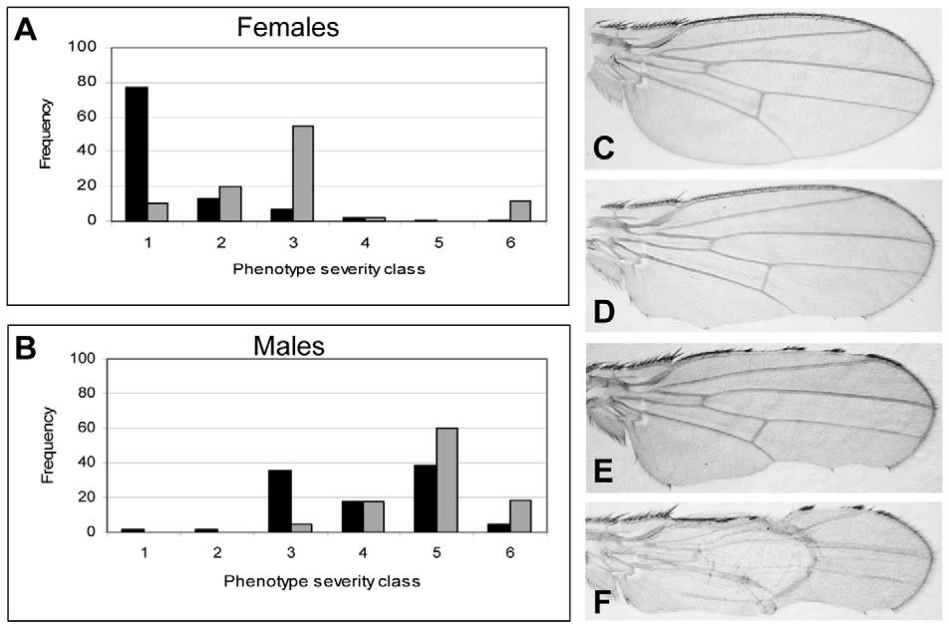
Genetic interaction between *Dlmo* and *ap*. (A,B) Female & male distributions of wing phenotypes. (C–F). Wings with various severities of *Dlmo^Bx2^* phenotypes (anterior side is up). (A) Genotype of the test group (in gray) is *Dlmo^Bx2^*/+; *ap^UGO35^*/+ and the control group (in black) is *Dlmo^Bx2^*/+. (B) Genotype of the test group (in gray) is *Dlmo^Bx2^*/Y; *ap^UGO35^*/+ and the control group (in black) is *Dlmo^Bx2^*/Y. Class 1 and (C), wild type wings; Class 2, one wild type wing and the other notched on the posterior side (D); Class 3, both wings are notched on the posterior side; Class 4, one wing is notched on the posterior side only and the other is notched on the anterior side as well (E); Class 5, both wings are notched on the posterior and anterior sides; Class 6 and (F), both wings are notched on the posterior and anterior sides and at list one wing also lacks dorsal to ventral adhesion. The distribution of the wing notching phenotype for the double heterozygous flies is shifted towards the more severe phenotypic groups.

*Dlmo^Bx^* mutants have been previously shown to genetically interact with various *ssdp* loss-of-function alleles [13]. Thus, the *Dlmo^Bx^* ^2^ allele provides a sensitized background to determine whether SSDP target genes function in D/V boundary formation. An example of the assay is depicted in Figure 3. Since *Dlmo* resides on the X chromosome, heterozygous females have a considerably less severe notching than hemizygous males (Figure 3). The wing notching phenotype displays a characteristic distribution of severities [12] allowing us to delicately determine the extent of genetic interactions by scoring enhancement or suppression of the wing notching phenotype by the Wilcoxon signed-rank test. The *Dlmo^Bx2^* wing phenotype was subdivided into six severity classes, where Class 1 represents flies with the least severe (wild type wings) and Class 6 represents the most severe wing notching. The control distributions were of *Dlmo^Bx2^*/+ females and *Dlmo^Bx2^*/Y males (Figure 3A and 3B, respectively). As expected, when the *Dlmo^Bx2^* mutation was combined with a heterozygous null mutation of *ap*, such as *ap^UGO35^* (*Dlmo^Bx2^*/+; *ap^UGO35^*/+ or *Dlmo^Bx2^*/Y; *ap^UGO35^*/+), the wing notching phenotype was enhanced, as evidenced by a shift of the distribution towards the more severe phenotypic classes in the double-heterozygous flies. Flies heterozygous for *ap^UGO35^* alone (*ap^UGO35^*/+) had normal wings. As expected due to the lack of *pnr* expression in this tissue, the *pnr* loss of function allele, *pnr^V1^*, did not interact genetically with *Dlmo^Bx2^* in our assay (Figure S1A and S1B).

If CHIP-AP transcriptional activity was synergistically reduced by mutations in *Dlmo* and *ap*, leading to the down-regulation of target genes of the CHIP-AP complex, then loss of function mutations in the target genes themselves (i.e. *Dlmo^Bx2^*/+; “*target gene* ^−^”/+ and *Dlmo^Bx2^*/Y; “*target gene* ^−^”/+) might have a similar effect on the *Dlmo^Bx2^* wing notching phenotype. This is indeed the case with *fringe* (*fng*), a known CHIP-AP target gene in the wing disc [38], which shows reduced expression in *Dlmo^Bx2^* mutant larvae dorsal wing pouch cells [9]. Double heterozygotes for *Dlmo^Bx2^* and *fng^80^* [38] exhibit a more severe wing notching phenotype than *Dlmo^Bx2^* alone, just as observed for the interaction of *Dlmo^Bx2^* and *ap^UGO35^* (Figure S1C and S1D). Control *fng^80^*/+ flies have normal wings.

We tested 39 genes from our original set of 189 SSDP candidate target genes in this genetic interaction assay with the *Dlmo^Bx2^* mutation (Table 1). These genes had publicly available mutant strains and their differential expression were evenly distributed (ranging between 0.00019 and 0.049 FDR-corrected p-values, Figure S2) in our array experiments. The mutations used were usually single transposable elements insertions, and where possible two independent mutant strains per gene were tested (allele-specific interactions are shown in Table S4). Strikingly, twenty eight of these genes (72%) interacted genetically with *Dlmo^Bx^* ^2^ (Table 1). This is a very high rate of agreement between the microarray results and the genetic interaction assay. In comparison we observed only 30% genetic interaction between *Dlmo^Bx^* ^2^ and a random set of 20 chromosomal deletions. These chromosomal deletions encompass 322 genes that are not included in the 189 SSDP target genes, such that the “background” interaction rate per gene is considerably less than 30%. These results indicate that a large number of the genes identified by the microarray are bona fide SSDP targets and have genetic functions in the CHIP-AP transcription complex pathway during wing development. As expected, most of the interacting target genes (25, i.e. 89%) enhanced the wing notching phenotype of *Dlmo^Bx2^* and only three (11%) suppressed it. In comparison, the interactions observed with the random set of deletions always suppressed *Dlmo^Bx2^*. Thus, loss-of-function mutations in SSDP target genes have a similar effect on *Dlmo^Bx2^* as loss of function mutations in *ap* and in its previously known target gene *fng*. This is consistent with a negative regulatory role for dLMO with respect to the CHIP-AP complex [7]-[9], [12], [14], [15]. SSDP target genes that failed to interact with *Dlmo^Bx2^* may be targets that are not dose sensitive, interact in different temporal or spatial contexts, or false positives.

**Table 1:** Genetic interactions between SSDP target genes and *Dlmo*, *ssdp* and *Chip*.

DGRC	Gene Symbol	*Dlmo^Bx2^*	*ssdp^L7^*	*chip^e5.5^*
CG10229	*katanin-60*	+	+	0
CG10236	*LanA*	+	0	−
CG11334	*CG11334*	−	n	n
CG11893	*CG11893*	+	+	+
CG12163	*CG12163*	+	+	0
CG12389	*Fpps*	+	0	0
CG12755	*l(3)mbn*	+	0	−
CG12800	*Cyp6d4*	+	+	0
CG14204	*CG14204*	+	−	0
CG1469	*Fer2LCH*	0	+	0
CG1518	*CG1518*	+	0	+
CG15489	*CG15489*	+	0	0
CG2604	*CG2604*	+	−	0
CG2674	*M(2)21AB*	−	+	−
CG2767	*CG2767*	+	+	0
CG2803	*CG2803*	0	n	n
CG2835	*G-salpha60A*	+	0	−
CG2986	*oho23B*	0	n	n
CG31689	*CG31689*	0	n	n
CG3186	*eIF-5A*	+	0	0
CG31991	*mdy*	+	0	0
CG3340	*Kr*	+	n	n
CG3488	*CG3488*	+	0	−
CG3725	*Ca-P60A*	0	n	n
CG4080	*CG4080*	+	0	+
CG4087	*RpP2*	+	0	−
CG4663	*CG4663*	0	n	n
CG4719	*BcDNA:LD22548*	+	−	−
CG4775	*l(2)k00619*	+	n	n
CG5431	*CG5431*	0	n	n
CG5446	*CG5446*	+	0	0
CG5725	*fbl*	0	0	0
CG6687	*CG6687*	0	+	0
CG6803	*CG6803*	+	+	+
CG7115	*BcDNA:LD21794*	0	n	n
CG7755	*CG7755*	0	−	−
CG7758	*ppl*	+	n	n
CG9415	*xbp1*	+	−	−
CG9932	*CG9932*	−	0	−

“+” Enhancer; “−” Suppressor”; “0” No interaction”; “n” Not tested”.

### *ssdp* and SSDP target genes interact genetically with *apterous*

**Figure 4:**
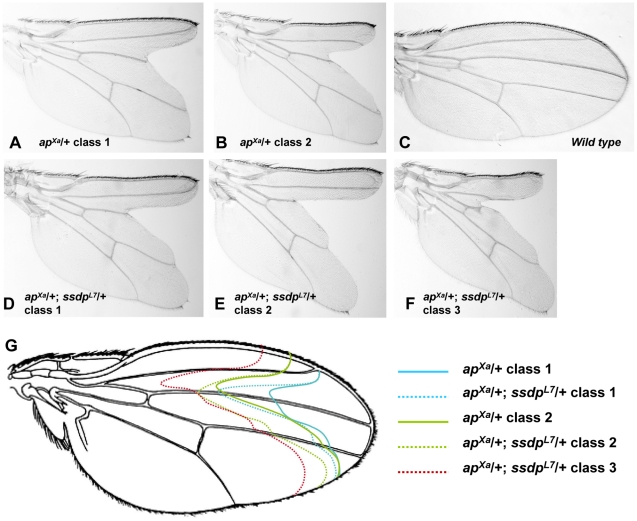
Genetic interaction between *ap^Xa^* and *ssdp^L7^*. (A,B) Wings of *ap^Xa^*/+ flies. (A) A typical class 1 wing. (B) A typical class 2 wing. (C) Wild type wing. (D–F) Wings of *ap^Xa^*/+; *ssdp^L7^*/+ flies. (D) A typical class 1 wing. (E) A typical class 2 wing. (F) A typical class 3 wing. (F) Schematic representation of the wing notching phenotypes depicted in (A–E). Classes 1–3 from *ap^Xa^*/+; *ssdp^L7^*/+ flies (represented by doted lines) are more severe then classes 1–2 from *ap^Xa^*/+ flies (represented by full lines).

Genetic interactions between *ssdp* and *Chip* or *Dlmo^Bx^* in a double heterozygous state are readily detected in the wing [13], but analogous genetic interactions between *ssdp* and loss of function alleles of *ap* are not. Therefore, to study the interactions between SSDP and CHIP-AP we needed another assay. We therefore explored using the only available dominant allele of *ap*, *ap^Xa^*, as a sensitized background. This mutant exhibits severe wing notching in a heterozygous state. We examined *ap^Xa^*/+ versus *ap^Xa^*/+; *ssdp^L7^*/+ flies, and observed augmentation of wing notching phenotype in the double heterozygous flies (Figure 4). In a population of *ap^Xa^*/+ flies, two classes of wing notching phenotypes can be distinguished (Figure 4A, 4B, and 4G) whereas the *ap^Xa^*/+; *ssdp^L7^*/+ flies exhibited three more severe wing notching classes (Figure 4D–4G). The *ap^Xa^* mutant is a gain of function allele [39], but its exact effect on the activity of the CHIP-AP complex is unknown. Our observation that *ap^Xa^*/+; *ssdp^L7^*/+ flies exhibit more severe wing notching than *ap^Xa^*/+ flies suggests that *ap^Xa^* causes reduced activity of the CHIP-AP complex, similar to *Dlmo^Bx2^*. These results clearly establish a genetic interaction between *ssdp* and *ap*, and indicate that *ap^Xa^* is useful for examining genetic interactions between candidate SSDP target genes and *ap*.

We tested seven of the SSDP target genes in the *ap^Xa^*/+ background (*ap^Xa^*/+; *target gene* ^−^/+). Mutations in the *katanin-60, CG12163* and *Myofilin* genes ameliorated the wing notching phenotype of *ap^Xa^*/+ flies, whereas *CG11893* and *Xbp1* mutations exacerbated wing notching (*CG1518* and *Cyp6d4* did not show an overt genetic interaction with *ap^Xa^*). These data indicate that both SSDP and SSDP target genes interact with AP and are therefore likely to act in a common pathway. Interestingly, the SSDP target genes enhanced the *ap^Xa^* wing notching less severely than *ssdp* itself, suggesting that the effect of SSDP is distributed among a large number of SSDP targets.

### SSDP target genes interact genetically with *ssdp* and *Chip* to form scutellar sensory bristles

The genetic interactions with *Dlmo^Bx2^* and *ap^Xa^* demonstrated that the SSDP target genes we identified are likely regulated by the CHIP-AP complex. Next we used genetic interactions to directly test our hypothesis that loss of SSDP disrupts the balance between the CHIP-AP and CHIP-PNR complexes in favor of the latter. To look at this balance between complexes, we examined thoracic bristles where both complexes function [16].

The CHIP-PNR complex positively regulates formation of thoracic sensory bristles via direct binding to the *ac/sc* enhancer. This CHIP-PNR function should be antagonized by AP since PNR and AP compete for binding of CHIP [16]. Consistent with our hypothesis, that loss of SSDP disrupts the balance between these two complexes, we found that both *ssdp^L7^* and *Chip^e5.5^* mutants display duplication of scutellar bristles as heterozygotes (<30% and <20% penetrance, for *ssdp^L7^*/+ and *Chip^e5.5^*/+, respectively, data not shown), a phenotype similar to gain of function alleles of *pnr* [35]. Flies heterozygous for *pnr^VX6^* alone have normal number of scutellar bristles. We therefore expected that double heterozygous flies (*ssdp^L7^*/+; *pnr^VX6^/+*) would exhibit reduced occurrence of scutellar bristle duplications due to the lower levels of *pnr*. Indeed, duplicated scutellar bristles phenotype was abolished in *ssdp^L7^*/+; *pnr^VX6^*/+ flies. Thus, reduced levels of *pnr* rescued the duplicated bristle phenotype of a loss of function *ssdp* mutant, supporting the antagonistic model for CHIP-PNR and CHIP-AP complex formation.

This model predicts that mutations in the SSDP target genes will have a similar phenotypic effect as altering the balance between CHIP-PNR and CHIP-AP complexes. To test this prediction we used the *ssdp^L7^* and *Chip^e5.5^* mutations as a sensitized background to screen the SSDP target genes for modifiers of scutellar bristle formation (*ssdp^L7^*/+; *target gene^−^*/+ and *Chip^e5.5^*/+; *target gene^−^*/+). Given the opposing roles of the CHIP-AP and CHIP-PNR complexes in this tissue we expected SSDP target genes to either enhance or suppress the duplicated scutellar bristles phenotype of *ssdp^L7^*/+ and *Chip^e5.5^*/+ depending on which of the two complexes regulates that particular SSDP target.

Mutations in twenty eight SSDP target genes were tested as double heterozygotes with either *ssdp^L7^* or *Chip^e5.5^* (allele-specific interactions are shown in Table S5). A total of 23 of them were found to interact with either *ssdp^L7^* or *Chip^e5.5^* (Table 1). Fourteen genes (52%) interacted genetically with *ssdp^L7^* and the same number of genes interacted genetically with *Chip^e5.5^*. Five genes (17.8%) interacted with both. This impressive rate of interaction suggests that SSDP targets are regulated by either or both CHIP complexes. The rate of interaction with CHIP and SSDP mutations in bristles is somewhat lower than what we observed for interaction with *Dlmo^Bx2^* in the wing. However, this is not surprising as SSDP target genes may be regulated by either AP or PNR or both, which might make bristles more robust to perturbation and thus make it harder to detect genetic interaction in the thoracic bristles compared with the wing, where only AP is present.

Among the 23 interacting SSDP target genes, mutations in 12 were found to partially suppress the duplicated scutellar bristle phenotype suggesting that they are positive regulators of scutellar bristle formation (Table 1). Conversely, mutations in 11 interacting SSDP target genes enhanced the duplicated scutellar bristle phenotype, suggesting that they are negative regulators of bristle formation (Table 1). Interestingly, ten of the 12 suppressors affected the *Chip^e5.5^* bristle phenotype and only five affected the *ssdp^L7^* bristle phenotype (three genes suppressed both *Chip^e5.5^* and *ssdp^L7^* phenotypes). In contrast, nine of the enhancers affected the *ssdp^L7^* bristle phenotype while only four enhanced the *Chip^e5.5^* phenotype (two genes enhanced both *Chip* and *ssdp* bristle phenotypes). Thus, it appears that loss of *ssdp* has a predominant effect on genes that negatively regulate scutellar bristle formation. This finding is consistent with our microarray and transcription factor binding site enrichment analyses which showed that loss of *ssdp* function resulted in down regulation of the CHIP-AP target genes, and with the antagonistic effect of AP on bristle formation. In contrast, although CHIP functions as a cofactor for both AP and PNR, the *Chip^e5.5^* mutation was more useful than the *ssdp^L7^* mutation for identifying genes that are positive regulators of scutellar bristle formation. The reason for this difference is unknown, but given the complexity evident when comparing the interactions and function of CHIP/LDB complex in just two tissues, it is likely that further complexity remains to be discovered in other contexts. The salient point is that our genetic interaction results demonstrate a clear modularity of the regulation of SSDP target genes by CHIP/LDB complexes in different tissues. Understanding this type of context-dependent component shuffling in transcription complexes will be required for a full understanding of transcriptional networks.

### Targeted silencing of SSDP target genes in *ap*- or *pnr*-expressing cells results in wing and thorax abnormalities

Our genetic screens described above tested the ability of heterozygous mutations in SSDP target genes to cause subtle changes in the dominant phenotypes of *Dlmo^Bx2^*, *ap^Xa^, ssdp^L7^* and *Chip^e5.5^* in the wing and scutellar bristles, respectively. Next we wished to determine whether the SSDP target genes identified are essential for proper development of these structures. The simplest way is to examine mutations in SSDP target genes in a homozygous state. Unfortunately, those mutations which were homozygous viable did not exhibit any wing or thorax morphological defects. For example, the *CG2604^EY05974^* mutation enhanced the *Dlmo^Bx2^* wing notching in a double heterozygous state (Table S4). Yet, in an otherwise wild type background, *CG2604^EY05974^* homozygous flies are viable and do not have any wing or thoracic morphological abnormalities (not shown). It is possible that these genes participate in, but are not essential for, wing and thorax formation, or that the mutations used to test for function were weak hypomorphs. For example, *CG2604^EY05974^*/*Df(3R)ED5147* exhibit ectopic wing veins (Figure S3) indicating that at least some of the failure to find homozygous mutant phenotypes is due the use of classic hypomorphic mutations.

Several of SSDP target gene mutations we used were homozygous lethal prior to adulthood, precluding examination of wing or thorax phenotypes. To avoid difficulties due to pleotyropic affects on viability, we utilized the transgenic GAL4/UAS system for targeted silencing of the SSDP target genes [40]. This approach offered two advantages: First, the UAS-RNAi constructs that were used are gene-specific. Second, expression of the UAS-RNAi can be targeted to a subset of cells depending on the GAL4 driver used while the rest of the cells maintain normal expression of the target gene, thus avoiding lethality. The *ap*-Gal4 [41] and *pnr*-Gal4 [35] drivers drive reproducibly high levels of UAS-lacZ transgene expression in cells known to express *ap* and *pnr* respectively, within the wing disc. Thus, by combining the transgenic constructs (*ap*-Gal4/+; UAS-RNAi-*target gene*/+ or *pnr*-Gal4/+; UAS-RNAi-*target gene*/+) we silenced SSDP target genes in either *ap*- or *pnr*-expressing cells. We knocked down nine SSDP target genes that interacted with *Dlmo^Bx2^*, *ap^Xa^, ssdp^L7^* and *Chip^e5.5^*. Silencing of two of them had profound effects.

**Figure 5:**
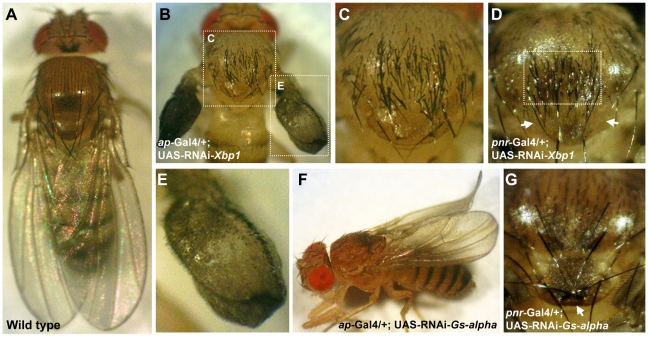
*Gs-alpha60A* and *Xbp1* are essential for normal wing and bristle formation. (A) Wild type fly. (B,C,E) *ap*-Gal4/+; UAS-RNAi-*Xbp1*/+. (B) Acute multiplication of bristles on the wing and scutum, wings are not fully developed and lack dorsal to ventral adhesion. White doted frames indicate areas enlarged in (C,E). (C) Enlargement of the thorax from (B), multiplication of bristles can be seen on the scutum but not on the scutelleum. (D) Thorax of a *pnr*-Gal4/+; UAS-RNAi-*Xbp1*/+ fly, multiplication of bristles is limited to the midline of the scutum, the area indicated by the white frame. The anterior pair of scutellar bristles are missing, arrows point to their expected position. (E) Enlargement of the wing from (B). Wings are underdeveloped and exhibit multiplication of bristles. (F) An *ap*-Gal4/+; UAS-RNAi-*Gs-alpha*/+ fly, wings are curled. (G) Thorax of a *pnr*-Gal4/+; UAS-RNAi-*Gs-alpha*/+ fly, the posterior pair of scutellar bristles indicated by an arrow are in reversed orientation.

Silencing of *Xbp1* (a.k.a. *CG9415*) in *ap*-expressing cells resulted in semi-lethality. Survivors reaching adulthood developed severely disrupted wings which appeared as small amorphic inflated structures, accompanied by marked excess of bristles on the wing and scutum, while the scutellum was not affected (Figure 5B, 5C, and 5E). As expected by the pattern of *pnr* expression in the adult fly [35], silencing of *Xbp1* in *pnr*-expressing cells caused a similar excess of bristles that were limited to the mid-line of the scutum while the wings were not affected. Interestingly, no extra bristles were observed on the scutellum, and some of the flies even exhibited a reduced number of scutellar bristles (Figure 5D). These observations indicate that *Xbp1* has opposing roles in regulating bristle development in the scutum and scutellum.

Silencing of *G-salpha60A* (a.k.a. *CG2835*) in *ap*-expressing cells caused a curled wing phenotype (Figure 5F). In addition, silencing of this gene in *pnr*-expressing cells reversed the orientation of the posterior pair of scutellar bristles (Figure 5G). It is therefore obvious that these two SSDP target genes are essential for normal wing and thorax development. The remaining seven SSDP target genes tested in this manner exhibited variable effects on the number of scutellar bristles and at very low penetrance. Given the large number of SSDP target genes and the likely robustness that this facilitates, some weak effects are expected. Combinatorial knock down experiments, much like the large set of double heterozyote tests we report here, will be required to piece these genes together into a more developed model. Importantly, like the CHIP-AP and CHIP-PNR complexes themselves, SSDP target genes show context-dependent effects on development.

## Discussion

*Drosophila* SSDP was identified on the basis of its ability to bind the nuclear adaptor protein CHIP/LDB [10], [13]. Both nuclear localization of SSDP [10] and its ability to modulate the transcription activity of the CHIP-AP complex during wing development [10], [13] depend on its interaction with CHIP/LDB. In the present study we have implemented a combination of molecular, bioinformatic and genetic approaches that allowed us to gain insight into the effect of SSDP on the transcriptional activity of CHIP/LDB complexes and their role in development. We have conducted a genome wide screen for SSDP target genes in *Drosophila* using expression microarrays with mRNA isolated from larvae bearing hypomorphic alleles of *ssdp*. Our analysis of transcription factor binding site enrichment served as an orthogonal assay that validates and extends the microarray results and thus contributes to our understanding of the relation between the CHIP-AP and CHIP-PNR transcription complexes in specific tissues (e.g. wing and thorax).

SSDP proteins directly bind DNA [29], and mouse SSDP1 activates the expression of a reporter gene in both yeast and mammalian cells indicating that it is capable of regulating transcription activity [21], [42]. We found enrichment for SSDP binding sites [29] upstream of the genes identified in the microarray experiments on flies lacking SSDP. Moreover, in agreement with the positive transcriptional role of SSDP, enrichment for SSDP binding sites was restricted to the genes showing decreased expression in mutants. This strongly suggests that a significant number of these genes are bona fide SSDP target genes.

Consistent with the involvement of SSDP with the CHIP-AP complex, we found that upstream regulatory regions of the SSDP putative target genes are also enriched for the AP binding site [31] and the SSDP binding site. These sites are likely to be functionally significant, since loss of *ssdp* enhances the wing notching phenotype of a dominant allele of *ap*. Additionally, over-expression of *Dlmo*, whose product negatively regulates the CHIP-AP complex, also interacts with mutants of SSDP target genes, demonstrating that SSDP target genes are involved in the CHIP-AP pathway. The efficiency of finding genetic interactions among the genes differentially expressed in the microarray experiments, demonstrated the power of this approach. Specifically, 72% of the loci we tested with *Dlmo^Bx2^* is more than an order of magnitude higher than an EP insertion screen (1.3% interacting) in a *Dlmo^Bx1^* sensitized background [43]. Our combined microarray and genetic loss of function screen allowed the identification of a similar number of *Dlmo*-interacting genes by screening a much smaller group of putative target genes [43]. Of the 35 genes identified by Bejarano and colleagues only *CG1943* was found in the 189 genes identified in our microarray screen. Our study specifically identified down-stream targets of SSDP, while those researchers searched for any modifiers of the *Dlmo* wing notching phenotype and thus uncovered genes that function in other regulatory pathways or genes that are upstream of the CHIP-AP complexes. This may explain the limited overlap between their results and ours.

In contrast to the enrichment of SSDP binding sites in the genes down-regulated in *ssdp* mutants we found the PNR binding site to be enriched specifically in the genes up-regulated in the *ssdp* mutants. We therefore present a model in which loss of SSDP disrupts the balance between the CHIP-AP and CHIP-PNR complexes. Mammalian SSDP proteins protect LDB, LHX and LMO proteins from ubiquitination and subsequent proteasome-mediated degradation by interfering with the interaction between LDB and the E3 ubiquitin ligase, RLIM. It is therefore possible that in the absence of SSDP proteins, CHIP/LDB and LMO can escape degradation by interacting with GATA and beta-HLH proteins that are not subjected to proteasome-mediated regulation [27]. The N-terminus of CHIP/LDB proteins is responsible for interaction with both PNR [16] and RLIM [22]. Thus, PNR/GATA proteins may partially interfere with the interaction between CHIP/LDB and RLIM making the CHIP/LDB-PNR/GATA complex more resistant to proteasome regulation and less dependant on the levels of SSDP proteins then the CHIP/LDB-LHX/AP complex.

According to our model, in cells where both the CHIP-AP and CHIP-PNR complexes are active, loss of SSDP should result in the same phenotype as over-expression of PNR. Indeed, we found that *ssdp^L7^*/+ flies display duplications of scutellar sensory bristles, similar to gain of function mutations in *pnr*. In addition, lowered levels of *pnr* in *ssdp^L7^*/+; *pnr^VX6^*/+ flies suppresses scutellar bristle duplication. This indicates that the duplicated scutellar bristle phenotype of *ssdp^L7^*/+ flies depends on the presence of PNR. As predicted by our model, since both AP and PNR regulate bristle formation, the functional interactions between SSDP target genes and *ssdp^L7^* and/or *Chip^e5.5^* resulted in either suppression or enhancement of the duplicated scutellar bristle phenotype.

Our results in flies indicate that SSDP contributes differentially to CHIP/LDB complexes containing AP versus PNR. By contrast, mouse SSDP proteins positively contribute to the transcription activity and assembly of both LDB-GATA and LDB-LHX complexes [13], [21], [22], [26], [27], [44], but the relative contribution of mammalian SSDP proteins to LDB complexes containing LHX proteins versus GATA proteins has not been specifically examined. It is possible that SSDP alters the balance of LIM-based CHIP/LDB complexes and GATA-containing CHIP/LDB complexes in the development of mice, as occurs in flies.

Our search for enrichment of transcription factor binding sites upstream of the putative SSDP target genes identified additional transcription factors that may warrant future study. Some of these factors are associated with SSDP and CHIP/LDB complexes. For example, the binding sites for PNR and ZESTE (Z) were both enriched in the up-regulated putative SSDP target genes. This is in agreement with previous studies showing that Z can recruit the BRAHMA (BRM, the *Drosophila* homolog of the yeast *SWI2/SNF2* gene) complex [45] via its member OSA [46], which together negatively regulate the CHIP-PNR complex during sensory bristle formation through direct and simultaneous binding of OSA to both CHIP and PNR [47].

Some of the additional regulatory inputs at SSDP target genes may be evolutionarily conserved. For example, we found enrichment of STAT92E and SSDP binding sites in the down-regulated SSDP target genes. This may be significant, as a known role of *ssdp* is regulation of the JAK/STAT pathway during *Drosophila* eye development [48]. Interestingly, mammalian STAT1 confers an anti-proliferative response to IFN-γ signaling by inhibition of c-*myc* expression [49]. Similarly, expression of mammalian SSDP2 in human acute myelogenous leukemia cells [50] and prostate cancer cells [51] leads to cell cycle arrest and inhibits proliferation accompanied by down-regulation of C-MYC. These findings indicate that both in *Drosophila* and in mammals SSDP and STAT proteins have similar functions and may share common target genes.

While our transcription factor binding site analysis utilized all of the 189 putative SSDP target genes, our genetic screens were conducted on a subset of them due to the availability of mutants. This suggests that more genetic interactions will be found among the untested genes. Even among this more limited subset, there are interesting new stories that suggest future experimental directions. For example, an insertion mutation in the *Xbp1* gene suppressed the duplicated scutellar bristle phenotype characteristic of *ssdp^L7^*/+ and *Chip^e5.5^*/+ flies, indicating that XBP1 contributes positively to bristle formation. In contrast, when *Xbp1* was silenced in *ap*-expressing cells both the wings and the scutum displayed a marked excess of sensory bristles while the scutellum was not affected. These results suggest that in the wing and scutum XBP1 acts as a negative regulator of bristle formation. Silencing of *Xbp1* in *pnr*-expressing cells caused a similar excess of bristle on the scutum, accompanied by a reduced number of scutellar bristles, further emphasizing the opposing effects of XBP1 in these two distinct parts of the thorax. Such contrasting phenotypes have been previously documented for several *pnr* mutants as well [35]. In flies and mammals XBP1 regulates the ER stress response, also termed the unfolded protein response (UPR, reviewed in [52], [53]). Since one of the functions of the ER is the production of secreted proteins, UPR-related pathways are widely utilized during the normal differentiation of many specialized secretory cells (reviewed in [52]). In this respect it would be interesting to examine whether SSDP and CHIP/LDB complexes affect the production of secreted morphogens, such as WINGLESS (WG), the secreted ligands of the EGFR receptor, SPITZ (SPI) and ARGOS (AOS), or the secreted NOTCH binding protein SCABROUS (SCA) (reviewed in [54]) via XBP1 during wing and sensory bristle formation. Alternatively, the transcription factor XBP1 may directly regulate the expression of genes required for differentiation of the wing and sensory bristles. Indeed, carbohydrate ingestion induces XBP1 in the liver of mice, which in turn directly regulates the expression of genes involved in fatty acid synthesis. This role of XBP1 is independent of UPR activation and is not due to altered protein secretory function [55]. Curiously, the two GO function categories ‘cellular carbohydrate metabolism’ and ‘cellular lipid metabolism’ which are enriched among *Xbp1* target genes in mouse skeletal muscle and secretory cells [56] were also enriched in our list of putative SSDP target genes (Table S6). Whether this reflects a secondary effect due to the down-regulation of *Xbp1* in *ssdp* mutants or a direct regulation of these processes by SSDP is yet to be determined.

Additional novel functions for CHIP/LDB complexes are implied by our results regarding the *Gs-alpha60A* (a.k.a. *CG2835*) gene. G protein coupled receptors are important regulators of development by for example, signaling via the protein kinase A (PKA) pathway [57]. Activation or inhibition of PKA signaling during pupal wing maturation perturb proper adhesion of dorso-ventral wing surfaces resulting in wing blistering. This phenotype may be due to miss-regulation of wing epithelial cell death [58] in *ap*-expressing cells [59]. Interestingly, similar wing blisters occur in the wing of *Dlmo^Bx2^* flies. Moreover, we found that mutant alleles of *Gs-alpha60A* enhanced the wing blistering phenotype of *Dlmo^Bx2^* (data not shown). Silencing of *G-salpha60A* in *ap*-expressing cells caused a curled wing phenotype. Such a phenotype can result from differences in the size of the dorsal and ventral wing blade surfaces. In addition, silencing of this gene in *pnr*-expressing cells caused the posterior pair of scutellar bristles to form in reversed orientation. Bristle orientation have been proposed to be regulated by planar cell polarity genes [60]. Taken together these results point to novel aspects of regulation of wing and sensory bristle development by SSDP and CHIP/LDB complexes mediated by G-alpha proteins.

### Conclusions

Our genome-wide expression profiling and bioinformatics analysis of *ssdp* mutant larvae, combined with genetic screens enabled us to gain insight into the intricate context-dependent transcriptional regulation by CHIP/LDB complexes. We were able to identify 28 putative SSDP target genes that are involved in wing development and 23 putative SSDP target genes that play a role in scutellar bristle formation. Examination of two of these, *xbp1* and *Gs-alpha60A*, suggests novel aspects of developmental regulation such as the involvement of SSDP and CHIP/LDB complexes in ER function and PKA signaling. Furthermore, we showed for the first time that SSDP proteins contribute differentially to transcription activity, and probably to the balance in formation of CHIP-AP and CHIP-PNR complexes. Furthermore we identified potential novel partners of SSDP in regulating transcription of downstream genes during fly development. It stands to reason that an extension of our genetic analysis to mammals and other vertebrates will reveal a host of additional functions of SSDP and CHIP/LDB during the multifaceted process of transcriptional regulation that underlies the development of multicellular organisms.

## Materials and Methods

### Fly handling

Unless otherwise stated, flies were grown on a standard medium containing cornmeal, yeast, molasses, and propionic acid at 25°C. The *ssdp* mutant strains (i.e *ssdp^BG1663^*, *ssdp^neo4^* ^8^ and *ssdp^31^*) used for the microarray experiment were previously described [13], all three were balanced on TM3-GFP (FBba0000338). The rev(*ssdp^neo48^*) line is a precise excision of the P element inserted in *ssdp^neo48^*. UAS-RNAi lines 18873, 38686, 38186, 24959, 24959, 6367, 40871, 9026, 12823 and 15347 were obtained from VDRC [61]. Chromosomal deletions Df(2L)ED49, Df(2L)ED548, Df(3L)ED231, Df(3L)ED4284, Df(2L)ED1109, Df(2L)ED299, Df(1)ED7067, Df(2R)ED2222, Df(3R)ED5156, Df(3L)ED4528, Df(2L)ED270, Df(2L)ED774, Df(2L)ED746, Df(3R)ED5187, Df(2L)ED673, Df(2L)ED120, Df(1)ED6957, Df(2L)ED19, Df(3R)ED5657 and Df(3R)ED10257, were obtained from the DrosDel collection [62]. All other fly stocks were obtained from the Bloomington Drosophila Stock Center (http://flystocks.bio.indiana.edu). Oregon-R flies were used as wild type.

#### Microarray

The transheteroallelic combination *ssdp^BG1663^*/*ssdp^neo48^* was obtained by crossing *ssdp^BG1663^*/TM3-GFP virgin females to *ssdp^neo48^*/TM3-GFP males. The trans-heteroallelic combination *ssdp^BG1663^*/*ssdp^31^* was obtained by crossing *ssdp^BG1663^*/TM3-GFP virgin females to *ssdp^31^*/TM3-GFP males. The control single heterozygotes *ssdp^BG1663^*/+ and *ssdp^31^*/+ were obtained by crossing virgin females from each mutant strain to wild type Oregon-R males. The control single heterozygote, *ssdp^neo48^*/+, was obtained by crossing virgin wild type females to mutant males. An additional control, *ssdp^BG1663^*/rev (*ssdp^neo48^*), was used instead of *ssdp^BG1663^*/+ for comparison to the trans-heteroallelic combination *ssdp^BG1663^*/*ssdp^neo48^* since they share more genetic background. The *ssdp^BG1663^*/rev (*ssdp^neo48^*) combination was obtained by crossing *ssdp^BG1663^* virgin females to rev(*ssdp^neo48^*) males. For detailed genotypes of microarray samples see Table S1. Crosses were made in vials containing colored medium: 7.5 g/l agar, 35 g/l flour, 50 g/l yeast, 55 g/l glucose, 2.5 ml/l p-Hydroxybenzoic Acid, 4 ml/l Propionic Acid and 0.5 ml/l Bromophenol. The colored medium allows for more precise staging of the larvae. Towards the end of the third larval stage the larvae cease to feed and the gut clears out. The colored medium can be seen through the live whole larvae. Larvae were collected when the gut was two thirds full and selected for the desired genotype using the GFP marker. Consequently only male larvae were taken for analysis to avoid artifactual differential expression due to sex biased expression in populations with different sex ratios.

#### Genetic interaction screen with *Dlmo^Bx2^*

Virgin *Dlmo^Bx2^* females were crossed to males harboring a mutation in a single target gene. Each cross was set up in 50 ml vials with 10 females and 7-10 males in each vial. All resultant F1 phenotypic classes were counted. The double heterozygote offspring (i.e *Dlmo^Bx2^*/+; *target gene^−^*/+ females and *Dlmo^Bx2^*/Y; *target gene^−^*/+ males) were counted according to their wing notching severity class. Class 1 representing flies with wild type wings; class 2 represents flies that have anterior notching of one wing; class 3 represents flies that have anterior notching of both wings; class 4 represents flies that have anterior notching of both wings and posterior notching of one wing; class 5 represents flies that have anterior and posterior notching of both wings and finally class 6 representing flies displaying partial detachment of the dorsal and ventral wing layers. Most crosses were set up in three vials and results were pooled. An average of 100 double heterozygote females and 114 double heterozygote males were counted for each target gene tested. The control *Dlmo^Bx2^*/+ females and *Dlmo^Bx2^*/Y males were obtained by crossing the *Dlmo^Bx2^* females to wild type Oregon-R males. A control cross was set up parallel to each set of test crosses. Rarely crosses were discarded if the control distribution was not consistent with previous control crosses. Finally, data from all the control crosses was combined to a single distribution for females and a single distribution for males and all the test distributions were compared to these two master controls. Significance was determined according to the Wilcoxon signed-rank test [63]. For 17 target genes more than one allele was tested (9 enhancers, 2 suppressors and 5 non-interacting). For a target gene to be designated as an interactor the same interaction was observed in both males and females. In addition where more than one allele was tested both alleles had to give the same interaction. If a target gene was tested by more than one allele and one allele gave the same interaction with males and females but the other only significantly affected one of the sexes it was still designated an interactor (2 enhancers and 1 suppressor). The DrosDel chromosomal deletions were compared to control *Dlmo^Bx2^*/*w^1118^* female and *Dlmo^Bx2^*/Y male flies obtained by crossing the *Dlmo^Bx2^* virgin females to males of the isogenic *w^1118^* line used to create the chromosomal deletions.

#### Genetic interaction screen with *ap^Xa^*

Virgin *T(2;3)ap* ^Xa^/In(2R)*Gla Bc Elp* females were crossed to *ssdp^L7^*/TM6- *Tb Sb e* males or males caring insertion mutations in different SSDP target genes. Each cross was set up in three 30 ml vials with 5 females and 3–5 males in each vial. The control *T(2;3)ap^Xa^*/+ flies were obtained by crossing the *T(2;3)ap* ^Xa^/In(2R)*Gla Bc Elp* females to wild type Oregon-R males. A control cross was set up parallel to each set of test crosses. Each test cross was compared to the control cross done in parallel. The resultant F1 of genotypes *T(2;3)ap^Xa^*/+, *T(2;3)ap^Xa^*/+; *ssdp^L7^*/+ or *T(2;3)ap* ^Xa^/+; *target genes^-^*/+ were counted according to their phenotypic severity class. Flies of genotypes *T(2;3)ap^Xa^*/+ or *T(2;3)ap^Xa^*/+; *target genes−*/+ exhibited two severity classes, class 1 being the least severe. Flies of the genotype *T(2;3)ap^Xa^*/+; *ssdp^L7^*/+ exhibited three severity classes which were different then those observed for *T(2;3)ap^Xa^*/+ or *T(2;3)ap^Xa^*/+; *target genes−*/+ flies and were therefore classified independently. Class 1 being the list severe and class 3 being the most severe.

#### Genetic interaction test with *ssdp^L7^* and *Chip^e5.5^*

Virgin *ssdp^L7^*/TM6-Tb Sb e or *Chip^e5.5^*/CyO-GFP females were crossed to males harboring a mutation in any single target gene. Each cross was set up in 50 ml vials with 10 females and 7–10 males in each vial. All resultant F1 phenotypic classes were counted. The double heterozygote offspring (i.e *ssdp^L7^*/+; *target gene^−^*/+ or *Chip^e5.5^*/+; *target gene^−^*/+) were counted and monitored for duplications of scutellar bristles. A control cross was set up parallel to each set of test crosses. The control *ssdp^L7^*/+ or *Chip^e5.5^*/+ flies were obtained by crossing the *ssdp^L7^*/TM6-Tb Sb e or *Chip^e5.5^*/CyO-GFP females to wild type Oregon-R males. Each test cross was compared to the control cross done in parallel. For the control cross the frequency of appearance of the duplicated bristle phenotype was calculated as p = (d+1)/(n+1) where n is the total number of flies and d is the number of flies displaying the duplicated bristle phenotype. Significance was determined using binomial cumulative distribution function with parameters p and m, m being the total number of flies in the test cross. The p-values calculated were corrected for multiple hypotheses testing using the false discovery rate procedure [64].

#### *In vivo* targeted RNAi silencing

The insertion alleles *ap^MD544^* and *pnr^MD237^* were used as *ap*-Gal4 and *pnr*-Gal4 respectively. Each cross was set up in three 30 ml vials with 5 females and 3–5 males in each vial and results were pulled. The control *ap*-Gal4/+ flies were obtained by crossing the *ap*-Gal4/In(2R)Gla Bc Elp females to wild type Oregon-R males. A control cross was set up parallel to each set of test crosses. Each test cross was compared to the control cross done in parallel.

### RNA procedures

RNA handling was performed exactly as described [65]. Briefly, larvae were flash frozen. Total RNA was extracted using Trizol (Life Technologies, Carlsbad, USA), followed by mRNA isolation using an Oligotex poly(A) extraction kit (Qiagen, Valencia, USA). RNA concentration was determined using RiboGreen dye (Molecular Probes, Oak Ridge, USA). RNA quality was determined by capillary electrophoresis using the 6000 Nano Assay kit (Agilent). All procedures were carried according to the manufacturer's instructions.

### Microarray

#### Data deposition

The FlyGem platform is available under GEO accession GPL20 [66] and the experiments described in this work are available under the series accession GSE20074. GEO sample accessions are given in Table S1.

#### Procedure

Microarray experiments were conducted exactly as described [65]. Briefly, samples were labeled with Cy3- or Cy5-labeled random nonamers (Trilink Biosciences, San Diego, USA). Hybridizations of samples to the microarrays were performed at 60°C, followed by washes. Arrays were scanned using an Axon GenePix 3000A fluorescence reader (Molecular Devices Corporation, Union City, USA). GenePix v.4.1 image acquisition software (Molecular Devices Corporation) was used to extract signal for each target element.

#### Statistical analysis

The array data was analyzed using R, which is an integrated suite of software facilities for data manipulation; calculation and graphical display (see http://www.r-project.org). The raw intensity data normalized within-arrays using the PrintTipLoess algorithm [67], and next between-arrays, using Quantile [68]. This normalization allows adjustment of microarray data according to effects that arise from variation in the technology rather than from biological differences between the RNA samples. Array elements whose intensity was lower than the median intensity in both channels were discarded. No other background correction method was used. We did not average duplicate array elements as this would reduce statistical power in later steps.

All 22 hybridizations were used to select for candidate target genes that were significantly differentially expressed between *ssdp* trans-heteroallelic combinations and their corresponding heterozygotes. An ANOVA fixed-model was used to determine significance. The p-value calculated was corrected for multiple hypotheses testing using the false discovery rate (FDR) procedure [64], [69], the threshold was set at <0.05.

### Analysis of enrichment of transcription factor binding sites

All genomic sequences were obtained from the UCSC genome browser (http://genome.ucsc.edu/, assembly Apr. 2006 for the *D. melanogaster* genome) [70]. The 500 bp upstream of the 189 candidate genes scanned using two algorithms termed PRIMA [30] and DEMON, for identifying enrichment of transcription factors binding sites in a set of co-regulated genes. Both methods require a background set for comparison (in this case all the annotated genes in *Drosophila*).

#### Transcription factors binding sites

The SSDP binding site was constructed from the linear sequence in Bayarsaihan et al, [29] and tested individually. The values in the matrix were set to 1 or 0 according to the appearance or nonappearance of the nucleotides in each position respectively. Those values were then corrected to allow flexibility in the recognition of this binding site. The binding site matrix for PANNIER was taken from [32] and tested together with all the binding site matrices available from the TransFac database (release 11.1) [31].The p-values calculated are corrected for multiple hypotheses testing using the false discovery rate procedure [64].

#### The PRIMA algorithm

Finds putative appearances of transcription factors binding sites in the promoters using a threshold score and then employs a hyper-geometric statistical test to examine whether those appearances are significantly over-represented in the data set with respect to the background set [30].

#### The DEMON algorithm

Based on hidden Markov models (HMMs) of promoter sequences regulated by a given transcription factor that take into account multiple binding sites of varying affinities in a promoter. DEMON builds an HMM for each one of the transcription factors binding sites and scores each pair of HMM-promoter for all the HMMs and the promoters in the data set. The score reflects how likely it is that the motif modeled by this HMM appears in this promoter. The scores are then utilized to obtain a p-value for each transcription factor binding site that reflects the probability that the binding site is enriched in the given set of promoters compared to a background set.

### Enrichment analysis of GO functions

Analysis for enrichment of GO functions was conducted using the **d**atabase for **a**nnotation, **v**isualization and **i**ntegrated **d**iscovery (**DAVID**) [71], [72]. Default setting were used and the enrichment cut off was set to p = 0.05 after FDR correction.

### Electrophoretic gel mobility shift assay

Fly *ssdp* was PCR amplified, cloned into pZEX plasmid and expressed with a GST tag in *E.coli* BL-21. Crude cell extract or purified GST-SSDP fusion protein was used for binding assays. GST-SSDP was purified on a glutathione agarose column (Sigma G4510). The *ssdp* single stranded CT oligonucleotide [29] was used as prob. Binding assays were carried out using the DIG Gel shift kit 2^nd^ generation (Roche, Mannheim, Germany) according to the manufacturer instruction in a final volume of 20 µl containing labeled DNA (150 fmoles), 1 µl of poly-L-lysine and 3 µl poly-[d(I-C)], 140 ng cell extract. For competition experiments 90 or 360 ng of unlabeled probe were added. Following a 20 min incubation at room temperature, the binding reaction products were separated on a native 6% polyacrylamide gel in 0.5% TBE (pH = 8). The gel was contact blotted onto a Hybond-N^+^ membrane (Amersham Biosciences). The chemiluminescent detection was performed following the manufacturer's instructions (Roche, Mannheim, Germany). The membrane was exposed to X-ray film (FUJI) for 15 min at 37°C.

## Supporting Information

Figure S1 Genetic interactions between *Dlmo* and *pnr* or *fng*. A&B *pnr^V1^* does not interact genetically with *Dlmo^Bx2^* in the wing. (A) Genotype of the test group (in gray) is *Dlmo^Bx2^*/+; *pnr^V1^*/+ and the control group (in black) is *Dlmo^Bx2^*/+. (B) Genotype of the test group (in gray) is *Dlmo^Bx2^*/Y; *pnr^V1^*/+ and the control group (in black) is *Dlmo^Bx2^*/Y. C&D *fng^80^* enhances the *Dlmo^Bx2^* wing phenotype. (C) Genotype of the test group (in gray) is *Dlmo^Bx2^*/+;*fng^80^*/+ and the control group (in black) is *Dlmo^Bx2^*/+. (B) Genotype of the test group (in gray) is *Dlmo^Bx2^*/Y; *fng^80^*/+ and the control group (in black) is *Dlmo^Bx2^*/Y.
(0.07 MB PPT)

Figure S2 The genes selected for the genetic interaction screen with *Dlmo^Bx2^* are evenly distributed. Each gene is plotted against the FDR transformed p-value generated by the ANOVA based statistical test used to determine the statistically significant genes that are differentially expressed between *ssdp* trans-heteroallels and their corresponding heterozygotes.
(0.03 MB PPT)

Figure S3 *CG2604^EY05974^* is a hypomorphic mutation. (A) Wild-type wing. (B) Wing of a *CG2604^EY05974^*/*Df(3R)ED5147* fly, ectopic wing veins are indicated by arrows.
(0.22 MB PPT)

Table S1 Sample genotypes of microarray hybridizations.
(0.02 MB XLS)

Table S2 SSDP putative target genes identified by microarray analysis.
(0.06 MB XLS)

Table S3 Transcription factors binding sites enriched in up stream regulatory sequences of SSDP target genes.
(0.07 MB XLS)

Table S4 Allele specific genetic interactions between SSDP target genes and *Dlmo^Bx2^*.
(0.02 MB XLS)

Table S5 Genetic interaction with *ssdp^L7^* and *Chip^e5.5^*.
(0.02 MB XLS)

Table S6 Biological proceses enriched in SSDP target genes as analysed by DAVID.
(0.02 MB XLS)
