# Supplementary material for: Transcriptional Regulation by CHIP/LDB Complexes
Source: PLoS Genet. 2010 Aug 12;6(8):e1001063. doi: 10.1371/journal.pgen.1001063 (PMC2921152; doi:10.1371/journal.pgen.1001063)
Supplement: Figure S1 — Genetic interactions between Dlmo and pnr or fng. A&B pnrV1 does not interact genetically with DlmoBx2 in the wing. (A) Genotype of the test group (in gray) is DlmoBx2/+; pnrV1/+ and the control group (in black) is DlmoBx2/+. (B) Genotype of the test group (in gray) is DlmoBx2/Y; pnrV1/+ and the control group (in black) is DlmoBx2/Y. C&D fng80 enhances the DlmoBx2 wing phenotype. (C) Genotype of the test group (in gray) is DlmoBx2/+;fng80/+ and the control group (in black) is DlmoBx2/+. (B) Genotype of the test group (in gray) is DlmoBx2/Y; fng80/+ and the control group (in black) is DlmoBx2/Y. (0.07 MB PPT) [file pgen.1001063.s001.ppt]

## Slide 1
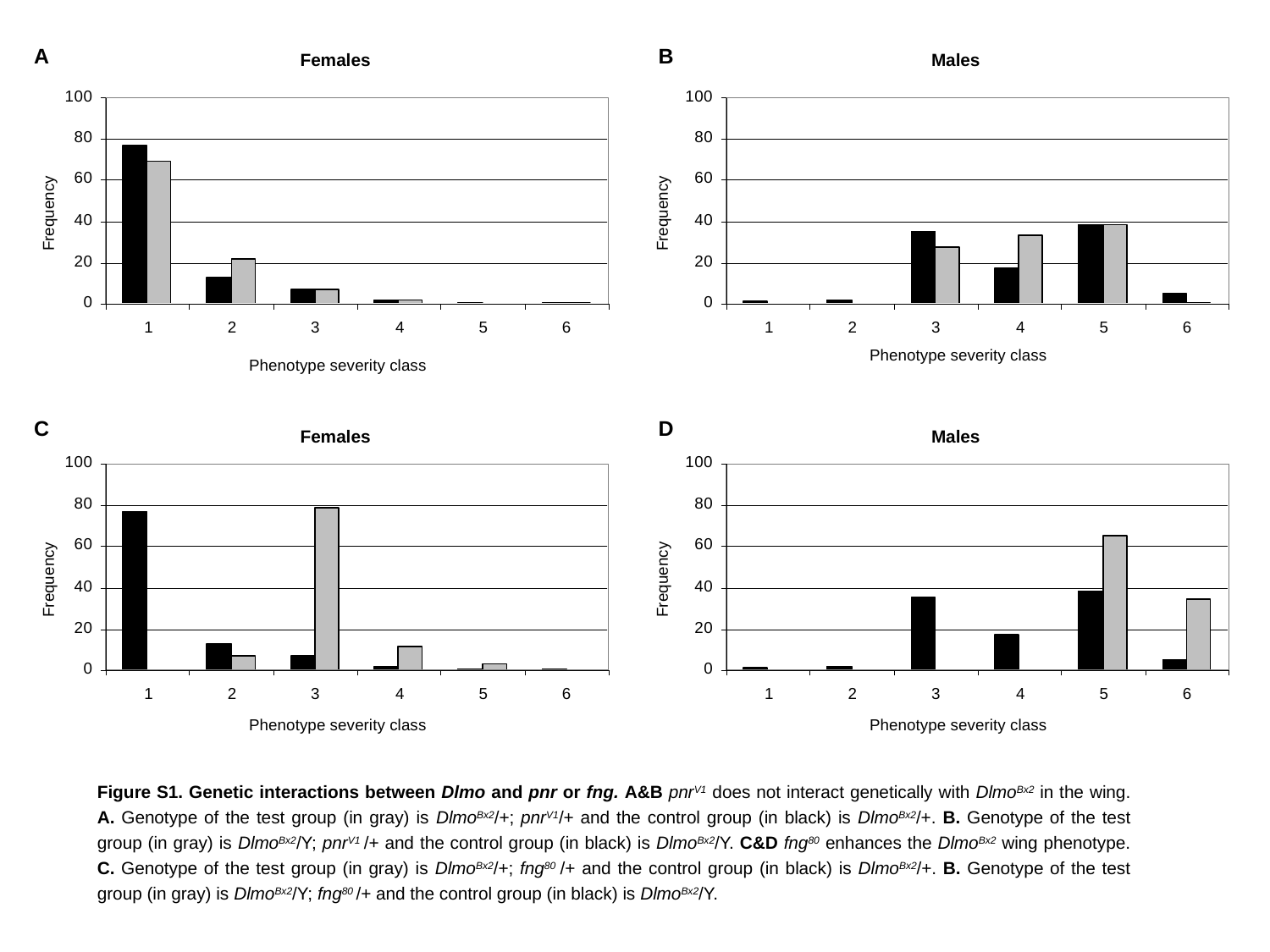

A
B
Females
Males
Frequency
Frequency
Phenotype severity class
Phenotype severity class
C
D
Females
Males
Frequency
Frequency
Phenotype severity class
Phenotype severity class
Figure S1. Genetic interactions between Dlmo and pnr or fng. A&B pnrV1 does not interact genetically with DlmoBx2 in the wing. A. Genotype of the test group (in gray) is DlmoBx2/+; pnrV1/+ and the control group (in black) is DlmoBx2/+. B. Genotype of the test group (in gray) is DlmoBx2/Y; pnrV1 /+ and the control group (in black) is DlmoBx2/Y. C&D fng80 enhances the DlmoBx2 wing phenotype. C. Genotype of the test group (in gray) is DlmoBx2/+; fng80 /+ and the control group (in black) is DlmoBx2/+. B. Genotype of the test group (in gray) is DlmoBx2/Y; fng80 /+ and the control group (in black) is DlmoBx2/Y.
