# Supplementary material for: Transcriptional Regulation by CHIP/LDB Complexes
Source: PLoS Genet. 2010 Aug 12;6(8):e1001063. doi: 10.1371/journal.pgen.1001063 (PMC2921152; doi:10.1371/journal.pgen.1001063)
Supplement: Figure S2 — The genes selected for the genetic interaction screen with DlmoBx2 are evenly distributed. Each gene is plotted against the FDR transformed p-value generated by the ANOVA based statistical test used to determine the statistically significant genes that are differentially expressed between ssdp trans-heteroallels and their corresponding heterozygotes. (0.03 MB PPT) [file pgen.1001063.s002.ppt]

## Slide 1
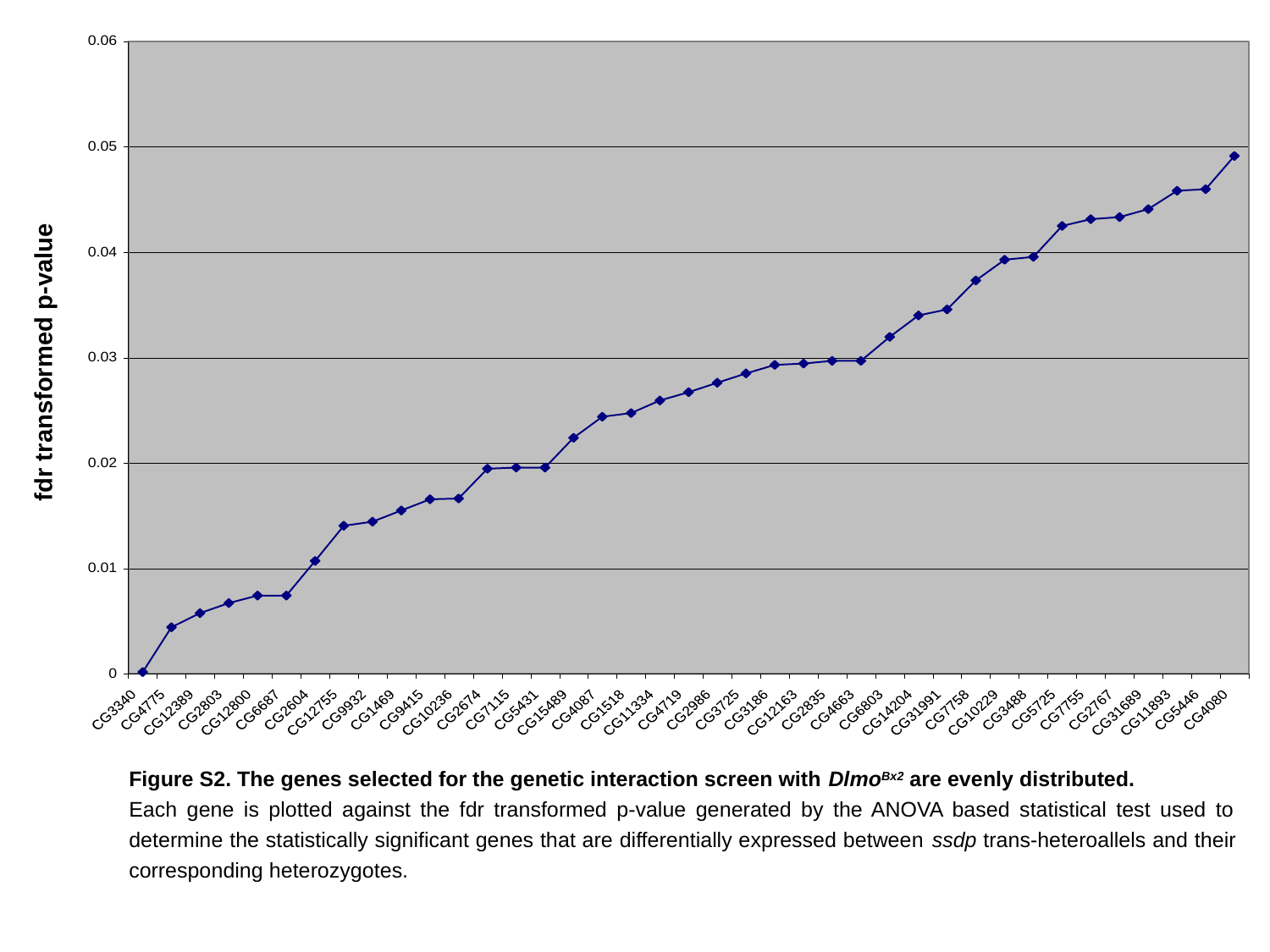

fdr transformed p-value
Figure S2. The genes selected for the genetic interaction screen with DlmoBx2 are evenly distributed.
Each gene is plotted against the fdr transformed p-value generated by the ANOVA based statistical test used to determine the statistically significant genes that are differentially expressed between ssdp trans-heteroallels and their corresponding heterozygotes.
