# Supplementary material for: Transcriptional Regulation by CHIP/LDB Complexes
Source: PLoS Genet. 2010 Aug 12;6(8):e1001063. doi: 10.1371/journal.pgen.1001063 (PMC2921152; doi:10.1371/journal.pgen.1001063)
Supplement: Figure S3 — CG2604EY05974 is a hypomorphic mutation. (A) Wild-type wing. (B) Wing of a CG2604EY05974/Df(3R)ED5147 fly, ectopic wing veins are indicated by arrows. (0.22 MB PPT) [file pgen.1001063.s003.ppt]

## Slide 1
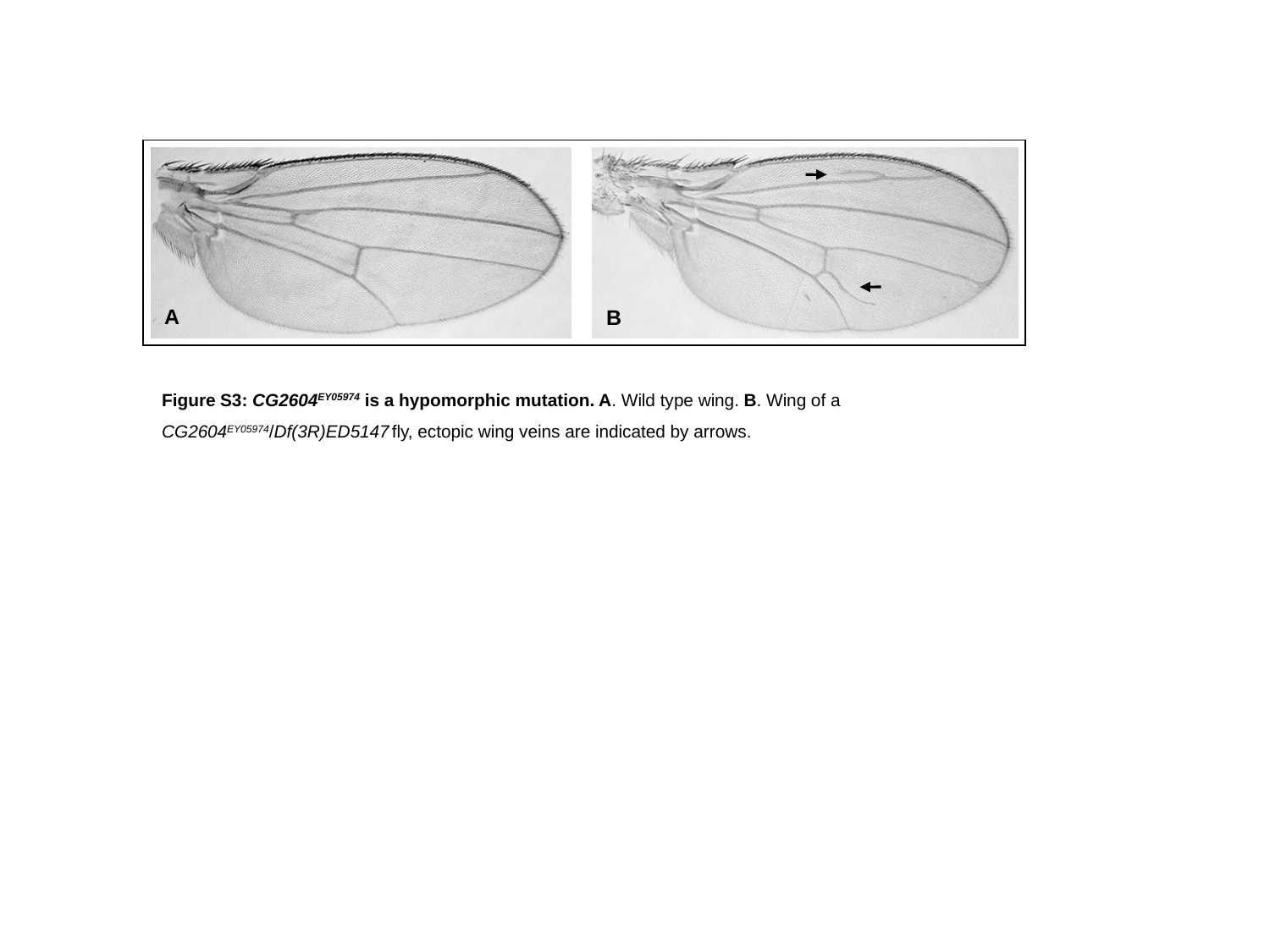

A
B
Figure S3: CG2604EY05974 is a hypomorphic mutation. A. Wild type wing. B. Wing of a CG2604EY05974/Df(3R)ED5147 fly, ectopic wing veins are indicated by arrows.
